# Fieldwork as a bridge between lectures and clinical clerkship: Medical students become the observer‐as‐participant

**DOI:** 10.1002/jgf2.468

**Published:** 2021-06-10

**Authors:** Kazuoki Inoue, Daisuke Son, Junko Iida, Shin‐ichi Taniguchi

**Affiliations:** ^1^ Department of Community‐based Family Medicine Faculty of Medicine School of Medicine Tottori University Yonago Japan; ^2^ Faculty of Health and Welfare Kawasaki University of Medical Welfare Kurashiki Japan

## CONFLICT OF INTERESTS

The authors have stated that there are no conflicts of interest in connection with this article.

To the Editor,

In this article, we report on a practical program in which medical students undertake participant observation in community medicine before clinical clerkship.

Since 2011, Tottori University Faculty of medicine has offered a community medicine practice to all fourth‐year medical students before clinical clerkship. The outline of this program is as follows: (1) orientation at the university, (2) clinical session in large‐ and medium‐sized hospitals and clinics (one day of practice per session for a total of four sessions in one month), (3) a summary workshop at the university, and (4) report writing and submission. We introduced an electronic portfolio (ePF) for feedback to the students. During this practice, we recognized the problem of not being able to encourage students to participate proactively. Therefore, in 2018, we started to engage the medical students in participant observation at community medical sites. Participant observation is a research method used in cultural anthropology in which researchers observe while participating in real social life.^1^

During orientation, we held a workshop on participant observation using photographs and video clip. Cultural anthropologists and medical educators collaborated on the orientation in which we focused on “making the strange familiar, and the familiar strange,” a saying from cultural anthropology.^2^

Raymond Gold has articulated four roles in social field observation.^3^ These roles are the complete participant, participant‐as‐observer, observer‐as‐participant, and complete observer. In the case of the complete participant, the subject is not aware that the fieldworker is conducting research. With the participant‐as‐observer, the researcher is given the role of an associate member, whereas the observer‐as‐participant conducts a one‐time visit to a site and conducts an interview or a survey. The complete observer role is where the researcher has no social contact at all with the subject.

**FIGURE 1:**
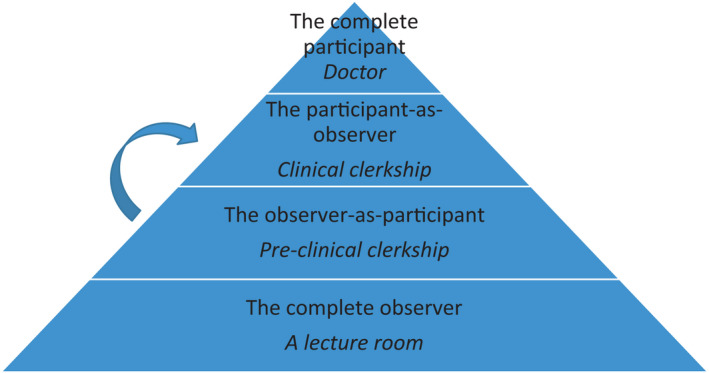
In this program which was conducted to pre‐clinical clerkship, medical students were placed in the role of the observer‐as‐participant. (Upright type font: Roles in participant observation, *Italic* *font*:*medical education*)

In our setting, we assumed that the complete participant was the doctor and complete observer was the medical student in the lecture room (Figure 1). During the orientation, we encouraged the students to participate as the observer‐as‐participant in the community medical field. The observer‐as‐participant's position has a stronger perspective as an observer than that of student doctor. In a study on the formation of doctors’ professional identity, it was found that medical students began to compare their observation of doctors with their preconceptions. They began to seriously consider doctors’ tasks, attitudes, and ways of thinking during clinical clerkship.^4^ By taking the step of the observer‐as‐participant, medical students may cultivate the viewpoints to verbalize what they observe and to reflect themselves during clinical clerkship. Therefore, this may help to shape a professional identity of medical students. Preliminary analysis of ePF showed that the most common words used in the student's descriptions were “observation” and “participation,” as well as “questioning” and “taking perspective.” From these results, it can be inferred that the students were able to formulate their own new questions based on their experience as the observer‐as‐participant.

Experiencing the observer‐as‐participant role in community medicine before clinical clerkship may help medical students approach their clinical clerkship from multifaceted perspectives and reconsider their medical careers.
